# Computer-aided diagnosis of colorectal polyps using linked color imaging colonoscopy to predict histology

**DOI:** 10.1038/s41598-019-39416-7

**Published:** 2019-02-27

**Authors:** Min Min, Song Su, Wenrui He, Yiliang Bi, Zhanyu Ma, Yan Liu

**Affiliations:** 1 Department of Gastroenterology and Hepatology, Affiliated Hospital of Academy of Military Medical Sciences, Beijing, 100071 China; 2 Pattern Recognition and Intelligent System Laboratory, School of Information and Communication Engineering, Beijing University of Posts and Telecommunications, Beijing, 100876 China

## Abstract

We developed a computer-aided diagnosis (CAD) system based on linked color imaging (LCI) images to predict the histological results of polyps by analyzing the colors of the lesions. A total of 139 images of adenomatous polyps and 69 images of non-adenomatous polyps obtained from our hospital were collected and used to train the CAD system. A test set of LCI images, including both adenomatous and non-adenomatous polyps, was prospectively collected from patients who underwent colonoscopies between Oct and Dec 2017; this test set was used to assess the diagnostic abilities of the CAD system compared to those of human endoscopists (two experts and two novices). The accuracy, sensitivity, specificity, positive predictive value (PPV) and negative predictive value (NPV) of this novel CAD system for the training set were 87.0%, 87.1%, 87.0%, 93.1%, and 76.9%, respectively. The test set included 115 adenomatous polyps and 66 non-adenomatous polyps that were prospectively collected. The CAD system identified adenomatous or non-adenomatous polyps in the test set with an accuracy of 78.4%, a sensitivity of 83.3%, a specificity of 70.1%, a PPV of 82.6%, and an NPV of 71.2%. The accuracy of the CAD system was comparable to that of the expert endoscopists (78.4% vs 79.6%; *p* = 0.517). In addition, the diagnostic accuracy of the novices was significantly lower to the performance of the experts (70.7% vs 79.6%; *p* = 0.018). A novel CAD system based on LCI could be a rapid and powerful decision-making tool for endoscopists.

## Introduction

Colorectal tumor is one of the most common tumors worldwide^1,2^. Adenomatous polyps are considered premalignant lesions of colorectal tumors^3^. Colonoscopies may play a significant role in reducing the incidence of colorectal tumors because they can detect and remove these adenoma polyps early. However, nearly 50% of small polyps prove to be non-adenomatous, and these polyps have no malignant potential^4^. The removal of a non-adenomatous polyp may exacerbate medical costs and cause many additional treatment risks, such as perforations, massive bleeding, and longer hospital stays^5^. Therefore, the accurate differentiation between adenomatous polyps and non-adenomatous lesions *in vivo* during colonoscopy is of significant clinical meaning.

Previous studies have reported that optical diagnoses may benefit the cost-effectiveness and efficiency of colonoscopies^5,6^. Moreover, recently, many advanced endoscopic image modalities have been developed to improve the differentiation ability in colorectal lesions, allowing for *in vivo* histological predictions. In particular, computer-aided diagnosis (CAD) based on narrow band imaging (NBI), which analyzes visually enhanced epithelial microvascular patterns, enables the prediction of polyp histology with a high accuracy rate^5,7–10^. However, an optical diagnosis using NBI to predict the histological results of polyps with high accuracy during colonoscopy requires training and expertise. Previous studies have reported nonacademic endoscopists could not achieve encouraging results using optical diagnosis^11,12^.

Linked color imaging (LCI), a new endoscopy modality, creates clear and bright images by using short wavelength narrow-band laser light. This modality can separately enhance the colors of lesions and make red areas appear redder and white areas appear whiter during a colonoscopy^13,14^. It may be possible to distinguish adenomatous and non-adenomatous polyps based on color evaluations of LCI images. Therefore, we developed a novel CAD system that analyzed LCI images of colorectal polyps to predict the histological results of these polyps. This CAD system was designed to assist endoscopists rapidly classify polyps as adenomatous or non-adenomatous regardless of expert or novice status. The aims of this study were to validate the diagnostic abilities of this CAD system and to compare the performance of the CAD system with that of experts and novice endoscopists in a pilot study.

## Methods

### Training of the CAD system

The algorithm for training the CAD system was based on a Gaussian mixture model (GMM) that consisted of four steps: (1) training data preparation; (2) parameter learning; (3) classification criteria creation; and (4) diagnostic output. The endoscope used in this study was a high-definition EC-L590ZW endoscope with the LASEREO system (Fujifilm Co., Tokyo, Japan), and all the examinations were performed by experienced endoscopists. Two experienced endoscopists reviewed LCI colonoscopy images from the picture archiving and communication system (PACS) database in our hospital and selected the regions of interest that contained the whole polyp in every image as the training set for CAD system. These images were classified as adenomatous or non-adenomatous polyps based on the histopathological results. The histological results were evaluated by three gastrointestinal pathologists to determine the type of polyp. During this process, poor-quality images were excluded. The exclusion criteria included out of focus images, dark images, blurred images and images with stained polyps. Finally, to train the CAD system, 139 images of adenomatous polyps and 69 images of non-adenomatous polyps were collected. No significant differences in age and sex were found between the adenomatous group and the non-adenomatous group.

**Figure 1:**
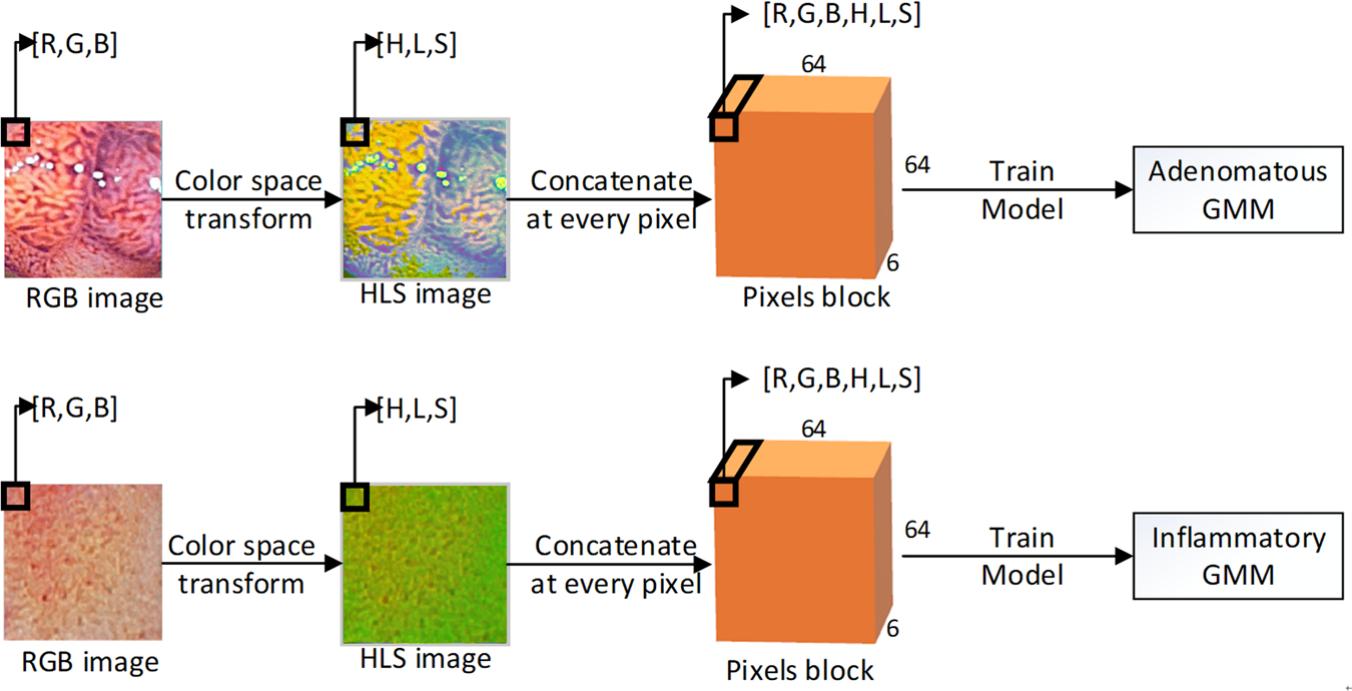
Flowchart of the training process. The original LCI picture is preprocessed to a 64 × 64 image where each pixel is represented as a 3-D vector (with R value, G value and B value). Transform the RGB color space to HLS. Now each pixel is represented as a different 3-D vector with H value, L value and S value. Concatenate 2 vectors to a 6-D vector. Do concatenation at every pixel of the image then we have a 64 × 64 × 6 pixels block. Feed these blocks into two initialized models separately to train for two independent GMMs.

**Figure 2:**
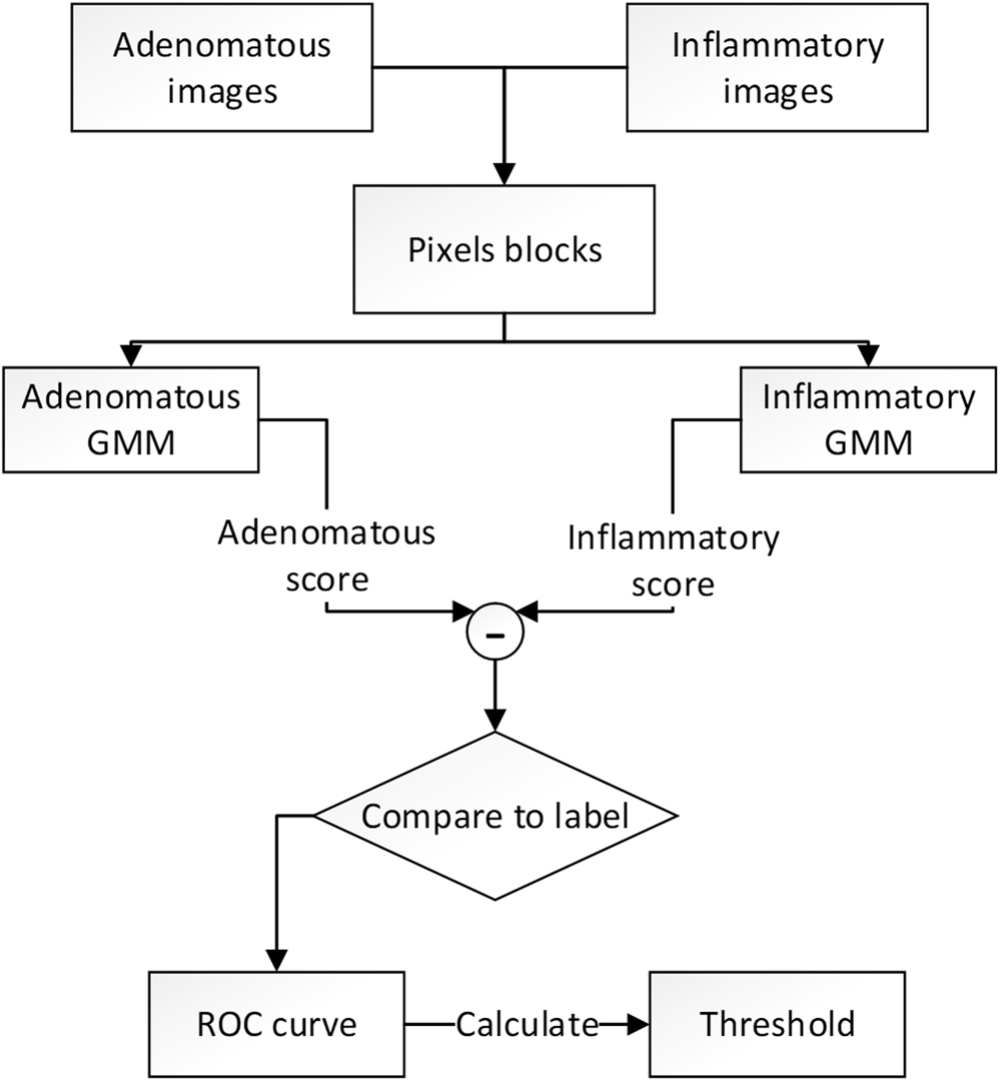
Flowchart of the threshold calculation. After training we obtain two GMMs. Transform all training images to pixels blocks and input the blocks to GMMs. We get two scores for each block consequently. Record the difference between the two scores of all blocks and plot the training ROC curve. Choose the point where the sensitivity is approximately equal to the specificity as the Threshold.

Our system was based on a GMM, which is usually used for clustering purposes. Given a set of unlabeled data, a trained model can attribute postulated sub-population identities to individual observations. However, it can also be implemented as a classifier using labeled data. In our work specifically, we trained a two-category classifier following the procedures (Figs 1 and 2):

1. Establish two GMMs, each of which contains 512 mixed components.
2. Preprocess images, including removing the background and resizing the images to a fixed size. Transform pixels from the RGB color space to the HLS space and then concatenate the RGB channels and HLS channels as a 6-D vector for each pixel.
3. Treat each pixel as a sample; there were 260,000 6-D vectors after eliminating the overexposed and underexposed pixels for each dataset.
4. Input the adenomatous dataset into one GMM to train the model and input the inflammatory dataset into the other model. After training, we obtained two trained GMMs.

**Figure 3:**
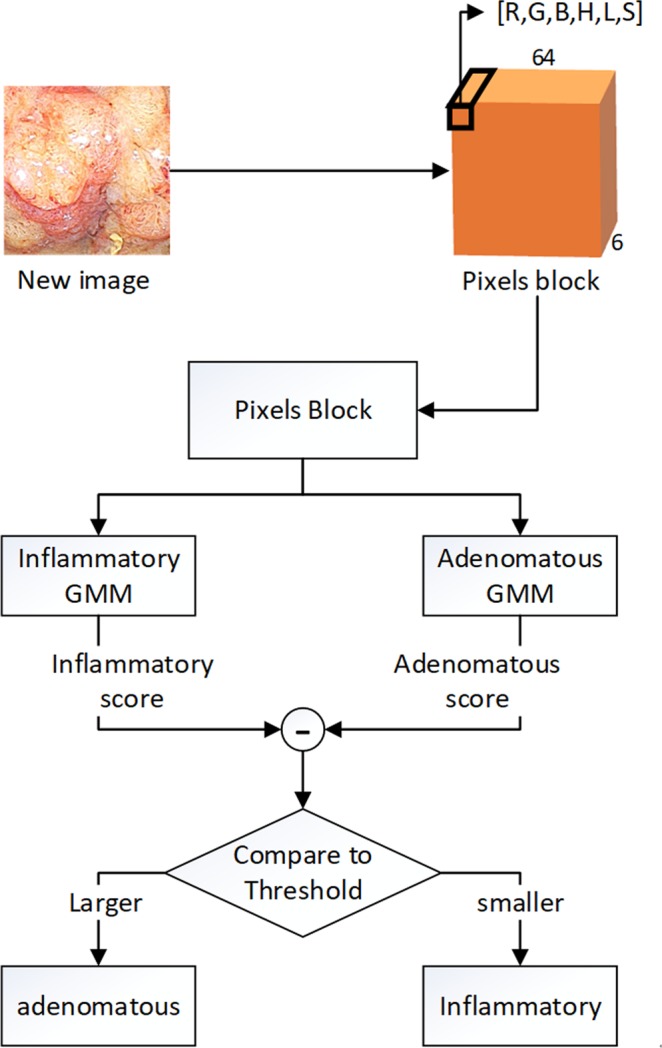
Flowchart of the test process. When a new image comes we transform it to pixels block and input it to two GMMs and obtain two scores. Get the difference between these two scores and compare it with the Threshold. If the difference is larger than Threshold, the image if classified as adenomatous image. Otherwise it is inflammatory image.

In the test stage, when a new image arrived, we preprocessed it into a set of 6-D vectors using the same procedure as step 2 and input these vectors into the two models independently, which produced two scores. If the difference between the adenomatous score and the inflammatory score was more than a pre-set threshold, the image was classified as an adenomatous polyp image. Otherwise, it was treated as inflammatory polyp image. The threshold was set such that the two training datasets had the closest (equal) training accuracies (Fig. 3).

### Comparisons between CAD and human endoscopists

#### Patients

After training the CAD system, we conducted a pilot study to prospectively compare diagnostic abilities among the CAD system, expert endoscopists and novice endoscopists. Consecutive patients who had planned colonoscopies at a tertiary hospital were screened for participation. Patients aged 16 to 80 years old were considered eligible if at least one polyp was found during their colonoscopy procedure. Exclusion criteria included patients who had inflammatory bowel disease, previous resection of the colon, familiar adenomatous polyposis, Peutz-Jeghers syndrome or other polyposis syndromes or conditions that impeded histological sampling, such as anticoagulant therapy, coagulation disorders or patient rejection. Patients undergoing colonoscopies were also excluded when the colonoscopy was not complete, the quality of the colon preparation was poor (Boston Bowel Preparation Score <4 points), colorectal cancer was detected or specimens were unavailable for histopathological assessment. All the included patients signed a written informed consent.

This study was approved by the Institutional Review Board of the Affiliated Hospital of the Academy of Military Medical Sciences according to the Consolidated Standards of Reporting Trials guidelines and was registered at ClinicalTrials.gov (study number: NCT03359343).

#### Image evaluations

All endoscopic procedures were performed by two experienced endoscopists. Total colonoscopies were completed in all patients. Polyps detected during colonoscopies were observed and captured with the LCI system. The location and size of all detected polyps were noted. The diameter of each polyp was estimated using biopsy forceps. The LCI images obtained for the test set were selected using the same image-quality criteria as the training set. Subsequently, another two expert endoscopists and two novice endoscopists, all of whom were blinded to histopathological results of the polyps, examined the LCI images of the test set and classified all included polyps as either adenomatous or non-adenomatous based on the LCI images alone.

All test-set images were exported to a hard drive in the same resolution as when examined by the endoscopists. Then, the CAD system analyzed each image and provided a histological prediction for the corresponding polyp.

#### Histopathology

The three expert gastrointestinal pathologists mentioned above evaluated all histological images of resected polyps using a routine histological protocol. The pathologists did not know the endoscopic diagnosis from either the expert endoscopists or the novice endoscopists. The pathological diagnosis of all resected polyps was considered to be the gold standard. For the purposes of analysis, because of the difficulty in diagnosing sessile serrated adenomas/polyps (SSA/Ps) and traditional serrated adenomas, these lesions were considered adenomatous.

#### Outcome measures

The primary outcome was the overall accuracy of the CAD based on LCI images in the differentiation of adenomatous and non-adenomatous polyps as well as the sensitivity, specificity, positive predictive value (PPV), and negative predictive value (NPV) of the system. The secondary outcome was the comparison of the accuracy, sensitivity, specificity, PPV and NPV among the CAD system, expert endoscopists and novice endoscopists.

#### Sample size

According to previous studies in which the NPV ranged from 73.5% to 85.2%^15–20^, the lower confidence level for specificity was set at >85%. A confidence level of 95% and a margin of accepted error of ±10% suggested a sample size of 58. In total, we collected 181 colorectal polyps from 91 patients for the test set.

#### Statistical analysis

A statistical software package (Statistical Package for the Social Sciences; SPSS Inc., Chicago, Illinois, USA) was used to perform statistical analyses. The mean and standard deviation (SD) were used to describe normally distributed data. The median was used to describe non-normally distributed data. The accuracy, sensitivity, specificity, PPV and NPV of the CAD system, expert endoscopists and novice endoscopists in the differentiation of polyps were assessed by comparisons with histopathology. Chi-square tests were used to compare proportions, and McNemar’s test was used to compare paired data. A two-tailed *P* value < 0.05 was considered statistically significant.

## Results

### Patient characteristics

Between Oct and Dec 2017, a total of 100 eligible patients signed informed consents for inclusion and underwent a colonoscopy in the Endoscopic Center of the Affiliated Hospital of the Academy of Military Medical Sciences. Based on the inclusion and exclusion criteria, a total of 9 patients were excluded. The characteristics of the 91 included patients are presented in Table 1.

**Table 1:** Characteristics of included patients (n = 203) and Polyps (n = 389).

	Training Set	Test Set
**Patients Characteristics**		
Patient number	112	91
male, n (%)	67 (59.8)	53 (58)
Age, mean ± SD, years	58 ± 8.9	56 ± 9.52
**Polyps Characteristics**		
Polyp number	208	181
No. of lesions per patient, median (IQR)	2 (1–5)	2 (1–6)
Size of lesion, mean ± SD, mm	7.8 ± 8.0	8.2 ± 7.9
Macroscopic type, n (%)		
Is	24 (11.5)	25 (13.8)
Isp	52 (25.1)	41 (22.7)
IIa	132 (63.4)	115 (63.5)
Location, n (%)		
Rectum	44 (21.1)	49 (27.1)
Sigmoid and Descending colon	41 (19.7)	44 (24.3)
Transverse colon	69 (33.2)	50 (27.6)
Ascending colon and Cecum	54 (26.0)	38 (21.0)
**Histopathology, n (%)**		
Adenomatous polyps	139 (66.8)	115 (63.5)
Non-adenomatous polyps	69 (33.2)	66 (36.5)

IQR, interquartile range; SD standard deviation.

### Polyp characteristics

A total of 217 polyps from 91 patients were included as the test set (Table 1). Of these polyps, 36 were excluded due to lost histopathology. Among the remaining 181 polyps, 38 (21.0%) were located in the cecum and ascending colon, 50 (27.6%) in the transverse colon, 44 (24.3%) in the descending and sigmoid colon and 49 (27.1%) in the rectum. According to the Paris classification of polyps, 25 (13.8%) of the polyps were of the protruded type (Paris Classification Is), 41 (22.7%) were of the sub-protruded type (Paris Classification Isp) and 115 (63.5%) were of the slightly elevated type (Paris Classification IIa). Histopathological evaluations showed that 66 (36.5%) of the polyps were non-adenomatous and 115 (63.5%) were adenomatous.

### Accuracy of CAD

**Figure 4:**
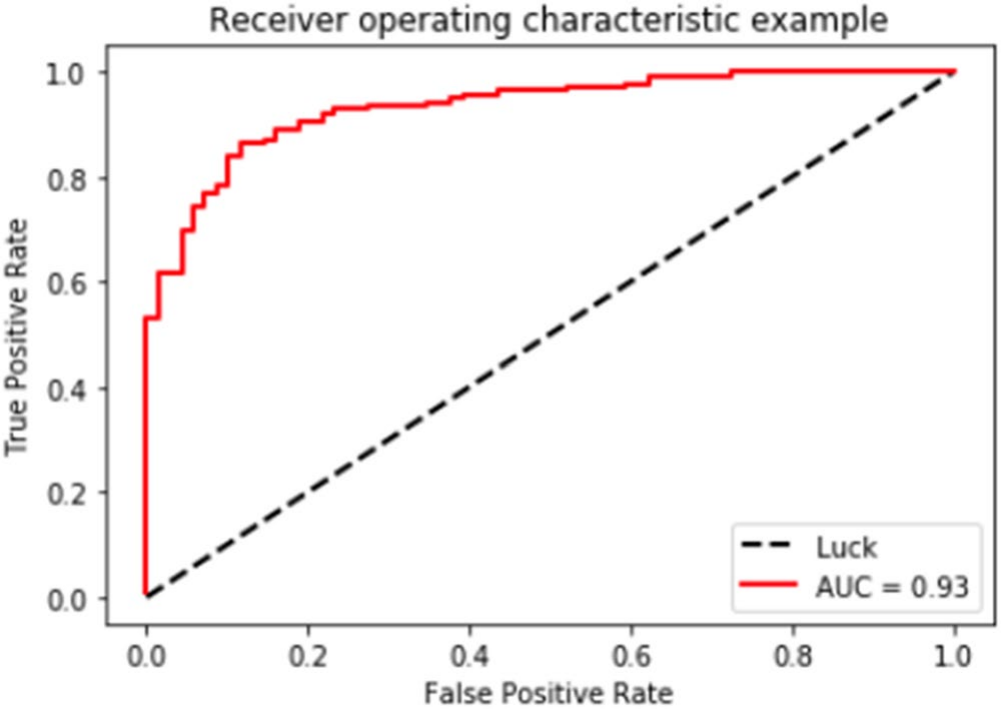
Receiver operator characteristic curve for the CAD differentiation of adenomatous versus hyperplastic polyps in the training set. AUC, area under the curve.

ROC curve for CAD performance in differentiating adenomatous from non-adenomatous polyps was calculated and the area under curve was 0.93 (P = 0.008) (Fig. 4). The accuracy of the CAD system was 87.0% (95% CI 60.7–81.7%) in the training set. The area under the curve was reasonably large with relatively high sensitivity and specificity. The diagnostic abilities of the CAD, expert endoscopists and novice endoscopists in the test set are presented in Table 2. The accuracy, sensitivity, specificity, PPV, and NPV of the CAD were 78.4% (95% CI 74.9–89.4%), 83.3% (95% CI 74.9–89.4%), 70.1% (95% CI 57.6–80.4%), 82.6% (95% CI 74.2–88.8%), and 71.2% (95% CI 58.6–81.4%), respectively.

**Table 2:** Diagnostic Performance of CAD and Endoscopists in Differentiating Adenomatous and non-adenomatous Polyps in the test set.

	CAD	Experts	Novice	P value	P value
				CAD vs experts	experts vs novices
Accuracy	78.4%	79.6%	70.7%	0.517	0.018
Sensitivity	83.3%	86.1%	74.8%	0.810	0.569
Specificity	70.1%	68.2%	63.6%	1.000	0.371
PPV	82.6%	82.5%	78.2%	0.733	0.065
NPV	71.2%	73.8%	59.2%	0.764	0.036

CAD, computer-assisted diagnosis; PPV, positive predictive value; NPV, negative predictive value; a: Percentages and 95% confidence intervals.

### Comparisons of the CAD, expert and novice endoscopists

The accuracy of the experts was 79.6%, and the novice endoscopists demonstrated an accuracy of 70.7%. Table 2 compares the diagnostic abilities of the endoscopists and the CAD. Among the 181 polyps, the CAD correctly classified 95 of the 115 adenomatous polyps (sensitivity of 83.3%) and 47 of the 66 non-adenomatous polyps (specificity of 70.1%).

## Discussion

Novel advanced imaging modalities should assist endoscopists in accurately differentiating adenomatous and non-adenomatous polyps because of the potential malignancy of adenomatous lesions. The removal of non-adenomatous polyps without malignant potential not only leads to the risk of procedure-related bleeding or perforations but also increases the costs and duration of the colonoscopic procedure and hospital stay^20^. However, for endoscopists to become familiar with the use of advanced imaging modalities requires intensive training, and interpretations of the findings remain subjective^16^. As the CAD results are objectively standardized, regardless of expert or novice status, the CAD can be useful, with little intraobserver or interobserver variability. This study, demonstrated that the LCI-CAD system has a promising diagnostic potential to predict the histopathology of colorectal polyps based on color analysis, with comparable accuracy to the eyes of human experts. Based on this system, endoscopists can easily distinguish adenomatous polyps from non-adenomatous lesions with good accuracies.

LCI using a laser endoscope (Fujifilm Co., Tokyo, Japan), a newly developed image-enhanced endoscopy (IEE), acquires images by using combined narrow-band short wavelength light and white light^21^. This novel endoscopic modality can enhance the color separation for red colors, which enables the reddish and whitish colors of lesions to become redder and whiter, respectively^14^. Our previous study and several other studies have demonstrated that the color processing in the LCI system can improve the visibility of colorectal polyps and predict certain kinds of gastrointestinal lesions^22–24^. When a polyp is adenomatous, the color is more likely to be deep red or purple whereas the color of a non-adenomatous lesion tends to be yellow or white. In our study, two experienced endoscopists obtained the images. The lesions from the gathered images were selected and extracted manually using obvious LCI features. The LCI-CAD system can analysis the LCI features of polyps and assist endoscopists in achieving accurate diagnoses by providing an objective judgment. Finally, in this study, the sensitivity of the CAD was 83.3%; the 17.4% of adenomas that were inaccurately differentiated using the CAD may be explained by the observational distance when we evaluated the lesions under the LCI model as well as yellow intestinal fluid covering the lesion due to inadequate bowel preparation, thus generating a false-negative analysis. This idea seems to be confirmed by the finding of lower accuracy for human endoscopists as well. We encountered some lesions that appeared yellow and blurred whose colors were redder and clearer after washing with water. These findings suggest that the appropriate distance and cleaning of lesions is particularly important for analysis.

In this study, the accuracy of the CAD was not high in comparison with the results of previous studies. The CAD system achieved an accuracy of 87.0% during the training process and 78.4% for the test set of polyps. ROC curve for differentiating the adenomatous from non-adenomatous lesion was calculated and it revealed that potential use of this CAD system for diagnoses. The area under the curve was quite large with sensitivity and specificity, which may be associated with the fact that a novel CAD system based on LCI could be a rapid and powerful decision-making tool for endoscopists,which could enlighten the future research. Other than the previously mentioned intestinal fluid covering the lesions, using only color as the single parameter included for analysis in the LCI-CAD system may partially explain the results compared with the results for the inclusion of capillary patterns and microstructure of the mucosa in NBI-based CAD analyses. Analyses based on the NBI system, which comprises the above two parameters, are more likely to have higher accuracy than analyses that only consider color for diagnoses^16,20,25,26^. Recent studies have mostly focused on magnification endoscopy or endocytoscopy^27^. In this study, not basing the analysis on magnification may be a strength of this CAD system; we excluded magnification as an additional parameter for analysis due to the following two reasons. First, acquiring high-quality magnified images during colonoscopy requires high-level steady movement of the endoscope, which can be particularly challenging for novice endoscopists and cause inevitable time costs. Second, in most regions in China and in Western countries, a magnifying endoscope is not commonly available in clinical practice^1^. However, LCI mainly focuses on the whole characteristics of polyps, specifically colors, with no need to maneuver to allow visibility of the microsurface or vascular patterns. However, to further improve the accuracy of diagnoses in the future, it may be possible to create a version of the CAD based on LCI with magnification or LCI with vascular patterns.

Another strength of our study is that a GMM was used to build the CAD system. With a relatively small LCI dataset (only 139 adenomatous LCI images and 69 inflammatory LCI images), it is very difficult to implement methods such as neural networks, which require large amounts of data. Compared with neural networks, a GMM is an easier and more explainable model that can process nonlinear data and obtain reasonable results with relatively small datasets^28^.

There are some limitations to this study. First, the training size was small because LCI is a novel endoscopic modality. As of this study, the building of a large database of LCI images is ongoing. To overcome this limitation, we applied a GMM, which is typically suitable for analyses of small size samples and provides accurate results. Second, this study was performed using still images rather than real-time evaluations of polyps. However, we believe the stable imaging of colorectal lesions in focus can be achieved during colonoscopy and analyzed in real-time practice in the future. Additionally, our CAD system performs at near real-time speeds (a 150-ms delay). Third, the incorporation of the CAD system into widespread clinical use is challenging. The incorporation of the CAD system into clinical endoscopic use should be well designed to ensure safety. Significant regulatory and reimbursement hurdles should be resolved before endoscopy integrated with a CAD system becomes a reality in clinical practice.

In conclusion, These findings may further broaden the spectrum of LCI clinical use in the future. Moreover, the novel CAD system developed based on LCI also demonstrates a promising performance and is comparable to the eyes of human experts. In particular, the dependence on non-magnified images makes this CAD system more easily accessible. We plan on conducting additional clinical trials to assess the potential use of this CAD system for real-time diagnoses. Additionally, further studies on this set of small and diminutive polyps may be designed to further investigate the performance of this CAD system.
